# The Role of Carotid Graft Interposition During Elective and Emergent Interventions: Protocol for a Scoping Review

**DOI:** 10.2196/91594

**Published:** 2026-09-25

**Authors:** Leonardo Pasquetti, Petar Zlatanovic, Edoardo Pasqui, Ognjen Kostic, Giuseppe Galzerano, Igor Koncar, Marko Dragas, Gianmarco de Donato

**Affiliations:** 1 Vascular Surgery, Department of Medicine, Surgery and Neuroscience, University of Siena, Viale bracci, Siena, 53100, Italy, 39 3316103816; 2 Clinic for Vascular and Endovascular Surgery/University Clinical Centre of Serbia, Belgrade, Serbia

**Keywords:** carotid artery, stenosis, endarterectomy, trauma, aneurysm, dissection, patch infection, internal carotid artery fibrosis, tumor, scoping review

## Abstract

**Background:**

In a significant proportion of carotid interventions, carotid graft replacement is required to achieve a successful outcome either as a primary method or as a bailout solution. Whether carotid grafting should be performed, the indications for its use, and other technical aspects remain insufficiently addressed in the literature. An exhaustive mapping of the sparse and heterogeneous evidence available in the literature may provide a more comprehensive understanding of this topic.

**Objective:**

This scoping review aims to examine and summarize the scientific evidence on the indications, graft types, and outcomes of graft interposition during elective and emergency carotid interventions.

**Methods:**

This scoping review will be conducted in accordance with the PRISMA-ScR (Preferred Reporting Items for Systematic Reviews and Meta-Analyses extension for Scoping Reviews) guidelines for reporting. Peer-reviewed papers written in English will be identified through searches of the following databases: PubMed (MEDLINE), Embase, Scopus, and Web of Science. The web-based systematic review platform Rayyan will be used for deduplication and study screening. The review will encompass the following clinical scenarios: elective carotid endarterectomy, emergent carotid endarterectomy, carotid artery restenosis, carotid artery trauma, carotid artery aneurysm, carotid artery dissection, carotid patch infection, radiation-induced carotid fibrosis, and carotid body tumors. All study designs (randomized controlled trials, observational studies, and case series) will be considered. Non-English studies, animal studies, cadaveric or anatomical-only reports, and purely technical notes without clinical data will be excluded. Data regarding extracranial-to-intracranial bypass will be excluded. Study selection based on title and abstract screening (first stage), full-text review (second stage), and data extraction (third stage) will be performed by a group of researchers, whereby each paper will be reviewed by at least 2 researchers. Any conflict regarding the inclusion or exclusion of a study and the data extraction will be resolved by discussion between the researchers who evaluated the papers; a third researcher will be involved if consensus is not reached. Data synthesis will be performed with the aim of clearly presenting the indications, graft types, and outcomes of graft interposition during carotid interventions.

**Results:**

The search strategy was finalized in July 2026. Preliminary searches of PubMed (MEDLINE), PROSPERO, and JBI Evidence Synthesis databases identified no published or registered systematic or scoping reviews specifically addressing carotid interposition grafting across elective and emergent carotid interventions. As of September 2026, formal database searches and study selection had not yet begun and were scheduled to start in October 2026. Data extraction and evidence synthesis are expected to be completed by spring 2027, and submission of the final scoping review is planned for summer 2027.

**Conclusions:**

Our scoping review will seek to provide an overview of the available evidence and identify research gaps regarding the role of graft interposition during elective and emergent carotid interventions.

## Introduction

### Background

Carotid disease encompasses a broad spectrum of medical and surgical conditions primarily affecting the carotid bifurcation, particularly the internal carotid artery.

The most prevalent entity is carotid atherosclerosis, which represents a major cause of stroke, mortality, and long-term disability worldwide [1]. When intervention is indicated, treatment of the atherosclerotic plaque can be achieved through either carotid endarterectomy (CEA) or carotid artery stenting (CAS), with the choice guided by patient-specific and anatomical factors. Carotid interposition grafting is a reconstructive technique involving replacement of a diseased or damaged carotid artery segment. It is reserved for complex clinical scenarios in which standard carotid reconstruction, including eversion CEA or conventional CEA with patch angioplasty, is not feasible or is unlikely to provide a durable repair. Beyond atherosclerosis, a heterogeneous group of less common carotid conditions may necessitate graft interposition.

Restenosis following carotid revascularization shows a variable incidence depending on the treatment modality and follow-up; early restenosis rates are modest, while long-term cumulative rates may be higher in selected series. The incidence ranges from 5% to 22% after CEA, with in-stent restenosis ranging from 2.7% to 33% [2]. A previous carotid procedure is a frequent indication for carotid interposition grafting [3]. Carotid trauma, both blunt and penetrating, is uncommon in general trauma cohorts but carries a high risk of neurologic morbidity when present and may require urgent vascular repair. Carotid artery injuries occur in approximately 6% of neck trauma cases and account for approximately 22% of vascular injuries [4]. Cervical artery dissection is a relatively infrequent cause of stroke in the general population (incidence of roughly 2.5‐5 per 100,000 person-years) yet accounts for a substantial proportion of ischemic events, especially in younger patients [5]. Extracranial carotid artery aneurysms are rare (generally reported as <1% of peripheral aneurysms and commonly cited in the 0.4%‐4% range of extracranial aneurysms), but when present, they pose risks of thromboembolism and rupture [6]. Although the type of aneurysm and the underlying anatomy influence the choice of repair, an open approach is recommended in the vast majority of cases [7].

Patch or prosthetic infections after CEA are rare complications that may require graft excision and complex arterial reconstruction [8]. Synthetic grafts or autologous vein grafts, harvested from the great saphenous or femoral vein, are commonly used [9]. Carotid body paragangliomas are rare, predominantly benign neuroendocrine tumors arising from the carotid body and are the most common type of head and neck paraganglioma, accounting for approximately 60%‐70% of cases [10]. Early surgical resection is feasible, but high Shamblin grade tumors increase the incidence of operative complications and the need for grafting [11]. Finally, prior neck irradiation produces radiation-induced arterial injury and perivascular fibrosis, with substantial reported rates of clinically significant carotid stenosis in survivors of head and neck cancer (estimates range up to approximately 20% within a few years in some cohorts) [12].

Collectively, these diverse etiologies present distinctive technical challenges that may necessitate carotid graft interposition rather than simple patch angioplasty or primary closure.

By replacing the diseased or damaged arterial segment with an autologous vein (commonly the saphenous or femoral vein) or a synthetic conduit (polytetrafluoroethylene or Dacron), carotid interposition grafting restores vessel continuity and ensures adequate cerebral perfusion.

Carotid graft interposition has been widely used over the years in both elective and emergent settings, although in a nonstandardized manner. Several gaps still exist regarding its indications, graft type selection, outcomes, and future scientific perspectives.

### Objectives

A comprehensive mapping of the sparse and heterogeneous evidence currently available in the literature may help clarify this clinically relevant topic. This scoping review aims to systematically collate and synthesize the existing evidence on carotid graft interposition in both elective and emergency settings, delineate the spectrum of clinical indications, techniques, and outcomes, and identify knowledge gaps to guide future research.

## Methods

### Guidance Framework

This scoping review will be conducted following the recommendations outlined by Levac et al [13] to guarantee a systematic and coherent process. The authors advocate proceeding by describing the following stages: (1) identifying the research question, (2) identifying relevant studies, (3) selecting studies, (4) charting and collating the data, and (5) summarizing and reporting the results. To report our findings, we will also adhere to the PRISMA-ScR (Preferred Reporting Items for Systematic Reviews and Meta-Analyses extension for Scoping Reviews) [14] checklist. A preliminary PRISMA-ScR flow diagram illustrating the planned study selection process is provided in Multimedia Appendix 1. Placeholder values are reported, and final numbers will be included in the completed scoping review.

### Stage I: Identifying the Research Question

The overarching research question guiding this scoping review is “What is the role of carotid graft interposition in elective and emergent carotid interventions with regard to clinical indications, surgical techniques, and outcomes?” To address this broad question, we formulated the following subquestions:

- In which clinical contexts has carotid graft interposition been reported (eg, restenosis, trauma, aneurysm, dissection, patch infection, tumor, and radiation-induced fibrosis)?
- What types of grafts (autologous vs synthetic) and surgical techniques have been described?
- What short- and long-term outcomes (eg, patency, stroke, mortality, restenosis, and complications) are reported?
- What knowledge gaps remain in the existing literature and what are the implications for future research?

These questions were structured according to the population, concept, and context framework to ensure clarity and comprehensiveness:

- Population: patients undergoing carotid interventions (elective or emergent)
- Concept: carotid graft interposition
- Context: clinical indications, surgical techniques, and reported outcomes in published studies

### Stage II: Identifying Relevant Studies

During the finalization of the search strategy in July 2026, preliminary searches were conducted in PubMed (MEDLINE), PROSPERO, and JBI Evidence Synthesis databases.

No published or ongoing systematic or scoping reviews addressing this specific topic were identified. Studies published from database inception to the date of the final search will be considered.

A comprehensive search strategy will be developed combining controlled vocabulary terms (MeSH/Emtree) and free-text terms related to *carotid graft interposition*, *endarterectomy*, *restenosis*, *trauma*, *aneurysm*, *dissection, fibrosis, neck tumor, and patch infection*. The detailed search is provided in Multimedia Appendix 2. In our final search, we will restrict our search to papers written in English and exclude gray literature. The search strategy will be reported in accordance with the applicable items of the PRISMA-S (Preferred Reporting Items for Systematic Reviews and Meta-Analyses literature search extension) guideline [15].

Rayyan will be used for reference management, deduplication, and study screening. Two reviewers will independently screen titles, abstracts, and full texts. Any disagreement will first be resolved through discussion and consensus between the 2 reviewers; if consensus cannot be reached, a third reviewer will adjudicate the decision. Data will be charted using a standardized extraction form developed by the review team.

A standardized extraction form will be used to capture study characteristics (author, year, design, population, indication, graft type, technique, and outcomes).

### Stage III: Study Selection and Eligibility Criteria

Studies reporting on carotid graft interposition in elective or emergent settings, with outcome data (patency, stroke, mortality, restenosis, and complications) will be included. All study designs (randomized controlled trials, observational studies, and case series) will be considered.

Non-English studies, animal studies, cadaveric or anatomical-only reports, purely technical notes without clinical data, and studies evaluating extracranial-to-intracranial bypass procedures will be excluded.

Two reviewers will independently screen titles, abstracts, and full texts. Discrepancies will be resolved by consensus or consultation with a third reviewer. Table 1 summarizes the inclusion and exclusion criteria.

**Table 1.** Eligibility criteria for the scoping review.

	Inclusion criteria	Exclusion criteria
Population	Adult patients (aged ≥18 y)	Young patients (aged <18 y), animal studies, and cadaveric- or anatomic-only studies
Concept	Carotid graft interposition (autologous or synthetic)	Procedures not involving carotid interposition grafting (eg, extracranial-to-intracranial bypass or other carotid reconstruction techniques without segmental arterial replacement)
Context	Clinical indications, surgical techniques, and reported outcomes (ie, patency, stroke, mortality, restenosis, and complications)	Clinical indications, surgical techniques, and outcomes not related to intervention (ie, carotid stenting outcomes without graft, medical therapy–only cohorts, and diagnostic imaging without intervention)
Setting	Hospital settings (vascular surgery) in elective or emergent conditions	Nonclinical or experimental settings
Study type	Original studies with any design or data type (quantitative and qualitative)	Other study types (eg, protocols, narrative reviews, or systematic reviews) or purely technical notes without clinical data
Publication status	Published in a scientific journal	Dissertations, books, conference papers, letters, and editorials
Publication language	English	Any language other than English
Full text	Full text available	Full text not available

### Stage IV: Charting the Data

The information from each selected publication, obtained from the final data extraction phase, will be summarized in a table.

Results will be mapped thematically according to indication (elective CEA, emergent CEA, restenosis, trauma, aneurysm, dissection, infection, tumor, and radiation-induced fibrosis) and outcomes (patency, complications, mortality, and stroke).

For each carotid graft indication (Table 2) the “indications,” “material and techniques,” and “outcomes and complications” sections will be explored (Textbox 1).

**Table 2.** Main clinical scenarios and indications for carotid interposition grafting.

Main scenarios	Indication
CEA^a^ reconstruction	Elective CEA Emergent CEA
Complications after CEA or CAS^b^	Restenosis after CEA Restenosis after CAS Patch infection Stent explantation
Other conditions	Neck tumor Carotid dissection Carotid trauma Carotid aneurysm Radiation-induced fibrosis

a CEA: carotid endarterectomy.

b CAS: carotid artery stenting.

Textbox 1. **Planned structure for reporting each clinical indication.**

Graft interposition during elective carotid endarterectomy

- Indications
- Materials and techniques
- Outcomes and complications
- Conclusions

### Stage V: Collating, Summarizing, and Reporting the Results

The extracted data will be synthesized using a descriptive and thematic approach, consistent with the objectives of a scoping review. No formal quantitative synthesis or meta-analysis will be performed.

Studies will first be grouped according to the primary indication for carotid graft interposition, including elective CEA, emergent carotid reconstruction, restenosis, aneurysm, trauma, dissection, patch or graft infection, tumor, and radiation-induced fibrosis.

Within each indication, studies will be summarized according to study characteristics (year, country, study design, and sample size), patient characteristics, indication for graft interposition, graft material (autologous vs prosthetic), surgical technique, and reported clinical outcomes.

The principal outcomes of interest will include technical success, perioperative stroke, mortality, graft patency, restenosis, graft infection, cranial nerve injury, reintervention, and any other procedure-related complications reported by the included studies.

Findings will be presented using summary tables and narrative synthesis. Frequencies and descriptive statistics will be reported whenever appropriate. The synthesis will specifically address each research question by mapping the indications for carotid graft interposition, the grafts and techniques used, the reported clinical outcomes, and the current gaps in the literature. A formal methodological quality or risk-of-bias assessment will not be performed, as the primary aim of this scoping review is to map the extent, characteristics, and gaps of the available evidence rather than to estimate intervention effects or determine the certainty of evidence. Nevertheless, the study design, sample size, and other key methodological characteristics of each included study will be extracted and reported.

## Results

The search strategy was finalized in July 2026. Preliminary searches of PubMed (MEDLINE), PROSPERO, and JBI Evidence Synthesis databases identified no published or registered systematic or scoping reviews specifically addressing carotid interposition grafting across elective and emergent carotid interventions. The final database searches are scheduled for October 2026. Title and abstract screening is expected to be completed by December 2026, followed by full-text assessment and data extraction between January and March 2027. Evidence synthesis and preparation of the final scoping review manuscript are expected to be completed by June 2027, with submission for publication planned for summer 2027.

## Discussion

### Clinical Insights

The clinical implications of carotid graft interposition are substantial, as this technique often represents the only viable option for restoring vessel continuity when standard reconstructive strategies are not feasible. The decision to use graft replacement is rarely trivial and depends on the underlying pathology, the urgency of presentation, and the available surgical resources. For example, in the context of carotid trauma or rupture of an extracranial aneurysm, timely graft interposition can be lifesaving and directly prevent catastrophic neurologic sequelae. In contrast, in elective scenarios such as restenosis, radiation-induced fibrosis, or tumor resection, the choice of graft type and technique may determine long-term patency, stroke risk, and durability of reconstruction.

A key clinical challenge is the heterogeneity of the conditions that may require grafting. Each entity—be it dissection, patch infection, aneurysm, or paraganglioma—presents distinct anatomical and pathophysiological considerations that influence technical feasibility and outcomes.

The choice between autologous and prosthetic grafts is not standardized and is often based on surgeon preference, institutional practice, and patient-specific factors. Consequently, outcomes vary widely across published series, and there is little consensus regarding optimal strategies in different disease contexts.

### Anticipated Contribution and Research Implications

This scoping review is expected to clarify the clinical scenarios in which carotid interposition grafting has been used, including complex CEA, restenosis after CEA or CAS, stent explantation, carotid aneurysm, trauma, dissection, patch infection, tumor resection, and radiation-induced fibrosis. The review will also map the autologous and prosthetic graft materials used, the reconstructive techniques described, and the reported perioperative and long-term outcomes.

By organizing the evidence according to indication, graft material, technique, and outcome, the review will identify areas in which clinical practice remains heterogeneous. Particular attention will be given to the absence of standardized criteria for selecting interposition grafting over alternative reconstructive strategies, the limited comparative evidence regarding autologous versus prosthetic conduits, and the inconsistent reporting of patency, stroke, cranial nerve injury, graft infection, restenosis, reintervention, and mortality.

The resulting evidence map may support the development of more consistent outcome definitions, inform the design of multicenter observational studies and registries, and help define clinically relevant questions for future comparative research. Although the review will not establish treatment recommendations, it may provide clinicians with a structured overview of the available evidence to support decision-making in complex carotid reconstruction.

### Limitations

Our selection criteria will limit inclusion to papers presenting empirical evidence published in English, which may bias the study pool toward research from Western countries. Consequently, readers should interpret the findings with caution, taking into account the variability in both quality and applicability of the included studies. The restriction to English-language publications will be adopted for feasibility reasons because resources for reliable translation and validation of non-English studies will not be available. Gray literature will be excluded to maintain a consistent minimum level of methodological and bibliographic information across included sources.

The subject heading “carotid graft” was selected for its relevance to the research scope. Subject headings belong to a controlled vocabulary that standardizes study indexing, whereas text words vary considerably depending on authors’ language choices. Such variability may influence the comprehensiveness of the search, as different terms can be used to describe similar concepts or the same term may carry distinct connotations across regions. Despite applying a rigorous approach and consulting experts, it remains possible that some relevant studies will not be captured, given the evolving terminology in this rapidly developing field.

This review will not assess the methodological quality of included studies, consistent with the objectives of scoping reviews. Heterogeneity in study design and outcome reporting is expected.

### Comparison With Previous Work

To the best of our knowledge, no systematic reviews or scoping reviews specifically addressing carotid graft interposition have been published to date. The existing body of literature on carotid interventions is dominated by studies on carotid artery stenosis, particularly in the context of endarterectomy and stenting, whereas evidence concerning other indications—such as carotid tumors, dissections, trauma, aneurysms, or patch infections—remains sparse and fragmented. Consequently, direct comparison with previous work is limited. The available reports outside of carotid stenosis are largely confined to small case series or isolated institutional experiences, which provide valuable clinical observations but lack generalizability. By consolidating this dispersed evidence, our scoping review seeks to fill an important gap in the literature and establish a structured foundation for understanding the role of carotid graft interposition across a wider spectrum of clinical scenarios.

### Conclusions

To the best of our knowledge, this scoping review will be the first to offer a comprehensive overview of the role of carotid graft interposition during elective and emergent interventions.

The findings of this scoping review will provide foundational knowledge on the role of carotid graft interposition, with particular emphasis on its indications, graft materials, and associated outcomes, across a broad spectrum of both common and rare carotid diseases. By identifying current evidence gaps and areas of clinical heterogeneity, the review may guide future research, improve clinician awareness, and support more informed decision-making regarding the optimal reconstructive approach.

## Supplementary material

10.2196/91594 Multimedia Appendix 1 PRISMA-ScR (Preferred Reporting Items for Systematic Reviews and Meta-Analyses extension for Scoping Reviews) flow diagram template for the planned study selection process.

10.2196/91594 Multimedia Appendix 2 Complete search strategy for the scoping review.

## Abbreviations

CAS: carotid artery stenting
CEA: carotid endarterectomy
PRISMA-S: Preferred Reporting Items for Systematic Reviews and Meta-Analyses literature search extension
PRISMA-ScR: Preferred Reporting Items for Systematic Reviews and Meta-Analyses extension for Scoping Reviews
