# Supplementary material for: The Role of Carotid Graft Interposition During Elective and Emergent Interventions: Protocol for a Scoping Review
Source: JMIR Res Protoc. 2026 Sep 25;15:e91594. doi: 10.2196/91594 (PMC13614217; doi:10.2196/91594)
Supplement: Supplementary file 1 [file resprot-v15-e91594-s001.docx]

**Multimedia Appendix 1: PRISMA-ScR Flow Diagram Template**

**Identification of studies via databases and registers**

Records identified from*:

- Databases:

Web of Science (n = to be determined)

SCOPUS (n = to be determined)

EMBASE (n = to be determined)

MEDLINE (n = to be determined)

- Other sources (n = to be determined)

**Identification**

Records after duplicates removed

(n = to be determined)

**Screening**

Reports excluded:

Reason 1 (n = to be determined)

Reason 2 (n = to be determined)

Reason 3 (n = to be determined)

etc

Reports screened (title/ abstract) (n = to be determined)

Reports excluded:

Reason 1 (n = to be determined)

Reason 2 (n = to be determined)

Reason 3 (n = to be determined)

etc

Full-text articles assessed for eligibility (n = to be determined)

Studies included in the scoping review (n = to be determined)

**Included**
