# Supplementary material for: The Role of Carotid Graft Interposition During Elective and Emergent Interventions: Protocol for a Scoping Review
Source: JMIR Res Protoc. 2026 Sep 25;15:e91594. doi: 10.2196/91594 (PMC13614217; doi:10.2196/91594)
Supplement: Supplementary file 2 [file resprot-v15-e91594-s002.docx]

**Multimedia Appendix 2: Search Strategy**

The complete electronic search strategy for PubMed/MEDLINE is reported below. The search strategy was developed using both Medical Subject Headings (MeSH) and free-text terms. The strategy was intentionally designed to maximize sensitivity in order to identify all studies reporting carotid artery interposition grafting or carotid reconstruction, irrespective of the underlying indication (e.g., atherosclerotic disease, restenosis, aneurysm, trauma, infection, tumour, radiation-induced fibrosis, or dissection). The search strategy will be adapted for Embase, Scopus, and Web of Science using the corresponding controlled vocabulary and syntax. No date or publication-type restrictions will be applied to the electronic search. The search will be limited to articles published in English. Animal studies, cadaveric or anatomical studies, pediatric populations, and reports without relevant clinical data will be excluded during study selection.

(

(

"Carotid Arteries"[Mesh]

OR "Carotid Artery Diseases"[Mesh]

OR carotid[tiab]

OR "carotid artery"[tiab]

OR "common carotid artery"[tiab]

OR "internal carotid artery"[tiab]

OR "external carotid artery"[tiab]

)

AND

(

"Vascular Grafting"[Mesh]

OR graft*[tiab]

OR "interposition graft*"[tiab]

OR "graft interposition"[tiab]

OR "carotid graft*"[tiab]

OR reconstruct*[tiab]

OR "carotid reconstruction"[tiab]

OR "vascular reconstruction"[tiab]

OR replacement[tiab]

OR "arterial replacement"[tiab]

OR "vein graft*"[tiab]

OR "autologous vein"[tiab]

OR prosthe*[tiab]

)

)

NOT

(

"Extracranial-Intracranial Bypass"[Mesh]

OR "extracranial-intracranial bypass"[tiab]

OR "EC-IC bypass"[tiab]

)
